# Population Genomic Analyses of the Sea Urchin *Echinometra* sp. *EZ* across an Extreme Environmental Gradient

**DOI:** 10.1093/gbe/evaa150

**Published:** 2020-07-22

**Authors:** Remi N Ketchum, Edward G Smith, Melissa B DeBiasse, Grace O Vaughan, Dain McParland, Whitney B Leach, Noura Al-Mansoori, Joseph F Ryan, John A Burt, Adam M Reitzel

**Affiliations:** Department of Biological Sciences, University of North Carolina at Charlotte; Department of Biological Sciences, University of North Carolina at Charlotte; Whitney Laboratory for Marine Bioscience, University of Florida, St. Augustine; Marine Biology Laboratory, Centre for Genomics and Systems Biology, New York University Abu Dhabi, Abu Dhabi, UAE; Marine Biology Laboratory, Centre for Genomics and Systems Biology, New York University Abu Dhabi, Abu Dhabi, UAE; Department of Biological Sciences, University of North Carolina at Charlotte; Marine Biology Laboratory, Centre for Genomics and Systems Biology, New York University Abu Dhabi, Abu Dhabi, UAE; Whitney Laboratory for Marine Bioscience, University of Florida, St. Augustine; Marine Biology Laboratory, Centre for Genomics and Systems Biology, New York University Abu Dhabi, Abu Dhabi, UAE; Department of Biological Sciences, University of North Carolina at Charlotte

**Keywords:** *Echinometra* sp. *EZ*, Persian/Arabian Gulf, RAD-seq, draft genome assembly, population dynamics, extreme environments

## Abstract

Extreme environmental gradients represent excellent study systems to better understand the variables that mediate patterns of genomic variation between populations. They also allow for more accurate predictions of how future environmental change might affect marine species. The Persian/Arabian Gulf is extreme in both temperature and salinity, whereas the adjacent Gulf of Oman has conditions more typical of tropical oceans. The sea urchin *Echinometra* sp. *EZ* inhabits both of these seas and plays a critical role in coral reef health as a grazer and bioeroder, but, to date, there have been no population genomic studies on this or any urchin species in this unique region. *E* sp. *EZ*’s life history traits (e.g., large population sizes, large reproductive clutches, and long life spans), in theory, should homogenize populations unless nonneutral processes are occurring. Here, we generated a draft genome and a restriction site-associated DNA sequencing data set from seven populations along an environmental gradient across the Persian/Arabian Gulf and the Gulf of Oman. The estimated genome size of *E*. sp. *EZ* was 609 Mb and the heterozygosity was among the highest recorded for an echinoderm at 4.5%. We recovered 918 high-quality SNPs from 85 individuals which we then used in downstream analyses. Population structure analyses revealed a high degree of admixture between all sites, although there was population differentiation and significant pairwise *F*_ST_ values between the two seas. Preliminary results suggest migration is bidirectional between the seas and nine candidate loci were identified as being under putative natural selection, including one collagen gene. This study is the first to investigate the population genomics of a sea urchin from this extreme environmental gradient and is an important contribution to our understanding of the complex spatial patterns that drive genomic divergence.

**Significance**

It is not yet well understood how population structure arises in marine systems, where there are generally few barriers to gene flow and organisms often have a high dispersal potential. In this study, we investigated the population genomics of an ecologically important sea urchin from the Persian/Arabian Gulf and the Gulf of Oman. We found that despite the high capacity for gene flow, there was still evidence of weak population structure. This may be a result of the extreme environmental gradient between the two seas. Further, we found that gene flow is likely bidirectional and characterized a collagen gene under putative selection. Thus, our study reveals how adjacent environments with opposing conditions could result in population genetic structure for ecologically important marine species with high dispersal potential.

## Introduction

The mechanisms governing evolutionary divergence are not well understood in marine systems, where clear barriers to gene flow are uncommon and organisms generally exhibit high dispersal capabilities (Palumbi et al. 1997; DeFaveri et al. 2013; Kelley et al. 2016; Oleksiak 2016; Takeuchi et al. 2020). Indeed, this propensity for dispersal coupled with large population sizes has been predicted to homogenize genetic variation and dampen the effects of genetic drift, respectively (Lande 1980; Waples 1998; Xuereb et al. 2018). Conversely, an increasing number of studies have shown signatures of genetic differentiation across small geographic scales as a result of oceanographic currents (Lal et al. 2017), organismal behavior that may favor local retention (Miller et al. 2001), and local adaptation due to environmental heterogeneity (Gleason and Burton 2016). Species with ranges spanning environmentally heterogenous ecosystems are highly informative study systems for advancing our understanding of complex population dynamics, as well as assessing the capacity of organisms for adaptation to changing environments (Reitzel et al. 2013; Gleason and Burton 2016).

The Persian/Arabian Gulf (hereafter PAG) is an example of an extreme marine environment, which is separated from the Gulf of Oman and the wider Indian Ocean by the narrow (42 km) Strait of Hormuz (Burt et al. 2019). The PAG is the world’s warmest sea with daily mean summer temperatures regularly >35 °C and extremes exceeding 37 °C (Burt et al. 2019; Smith et al. 2017b). These conditions surpass climate change predictions for the Indo-Pacific in the next century (Hoegh-Guldberg et al. 2014). The neighboring Gulf of Oman (hereafter GO) experiences much lower summer temperatures which are typically <32 °C (Coles 2003). In addition to extreme thermal conditions, the PAG also experiences higher salinity than the GO (40–42 PSU vs. 37 PSU, respectively) (Burt et al. 2008; Bauman et al. 2013). To date, several studies have shown population structuring between the two seas, including in the brain coral, *Platygyra daedalea* (Howells et al. 2016; Smith et al. 2017a), the sea urchin *Diadema setosum* (Lessios et al. 2001), and several species of fishes (Hoolihan et al. 2004; Giles et al. 2014; Torquato et al. 2019). The majority of studies in this region focused on corals and fishes (Vaughan and Burt 2016) and the only genetic work on sea urchins used short mitochondrial sequences. To better understand these diverse ecosystems, it is crucial to examine species with different life history traits (i.e., population size, dispersal patterns, mating systems, philopatry, and reproductive timing) as these traits may mediate the effects of gene flow and genetic drift (Whiteley et al. 2004).

Sea urchins are critical ecosystem engineers around the world, particularly in shallow coastal habitats, where their grazing plays an important role in bioerosion and algal control (Downing and El-Zahr 1987; McClanahan and Muthiga 2007). Due to their importance, urchins have been used as study systems to determine the potential for adaptation to stressful environmental conditions (Pespeni et al. 2012, 2011; Kelly et al. 2013; Pespeni and Palumbi 2013). To date, there have been no population genomic studies of any species of sea urchin within the entire northeastern Arabian region (although there have been population genetic studies based on mitochondrial regions, see: Bronstein and Loya, 2013 and Lessios et al. 2001). This represents a crucial knowledge gap as sea urchins are highly abundant in the PAG (densities averaging 8.6 m^−2^ across eight sites between 2015 and 2019; Burt JA, unpublished data) and they play a significant role in the health and dynamics of coral reef ecosystems in the region as major bioeroders (Downing and El-Zahr 1987). The most abundant sea urchin in the PAG is *Echinometra* sp. *EZ*, previously thought to be *Echinometra mathaei* (Ketchum et al. 2018)*. Echinometra* are common in shallow water (1–3 m depth) and have been found in waters up to 20 m deep (McClanahan and Muthiga 2007). The seasonal reproductive patterns of regional *Echinometra* sea urchins are not yet well understood; however, one study in the northern PAG showed peak spawning in June (Alsaffar and Lone 2000). The larvae feed on phytoplankton and although the pelagic larval duration (PLD) for this species is unknown, congeners have PLDs of a few weeks (e.g., *Echinometra vanbrunti* [18 days] and *Echinometra viridis* [30 days]; McClanahan and Muthiga 2007). On a large spatial scale, these larvae behave as passive particles and their transport is governed by oceanographic current patterns (Rahman et al. 2014). Taken together, these traits make *E*. sp. *EZ* an excellent study organism to better understand how different life history strategies can drive molecular evolution, resulting in different patterns of population divergence in the PAG.

In this study, we used restriction site-associated DNA sequencing (RAD-seq) to characterize patterns of genetic diversity and population structure of *E.* sp. *EZ.* We collected samples from seven sites spanning >500 km from the southern PAG into the western GO. We generated a draft genome to use as a reference for our RAD-seq analyses. We performed an initial outlier analysis to identify SNPs under potential selection and characterized historical gene flow to understand migration patterns. This study contributes to our understanding of genetic differentiation in marine invertebrates in environmentally divergent habitats and how this may pertain to a changing climate.

## Materials and Methods

### Draft Genome Assembly

#### Sample Collection.

A gonadal tissue sample from a single *E*. sp. *EZ* adult from Dhabiya reef in the southern PAG (24°21′55.8″N 54°06′02.9″E) was collected, preserved in RNA*later*, and subsequently stored at −20 °C.

#### DNA Extraction and Sequencing.

Total genomic DNA was extracted from the gonadal tissue sample using the DNeasy Blood and Tissue Kit (Qiagen). DNA quality was visualized on an agarose gel and concentration was determined with a 2000 Nanodrop spectrophotometer (ThermoFisher Scientific, Waltham, MA). High-quality DNA was submitted for PCR-free library preparation and whole-genome sequencing on one lane on an Illumina HiSeq3000 (150-bp paired-end reads) and one lane on a NextSeq500 (150-bp paired-end reads) at the University of Florida Interdisciplinary Center for Biotechnology Research.

#### DNA Read Processing and Genome Assembly.

Approximately 302 million paired-end (PE) reads were obtained from the HiSeq and NextSeq sequencing. We performed adaptor trimming and quality filtering using Trimmomatic v0.36 (Bolger et al. 2014) with a phred quality score of 33. Leading and trailing bases with a quality score <3 were removed, a 4-base wide sliding window was used to cut where the average quality per base dropped <15, and reads that were <36 bp long were removed. This was followed by error correction with Allpaths-LG version v44,837 (Gnerre et al. 2011). To estimate genome size, we generated a frequency histogram for a *k*-mer length of 21 using Jellyfish v2.2.6 (Marçais and Kingsford 2011). This histogram was then analyzed using GenomeScope to obtain estimates for genome size, as well as heterozygosity and duplication levels (Vurture et al. 2017). Mitochondrial reads were removed using FastqSifter v1.1.1 (Ryan 2015a) with the *E. mathaei* mitochondrial genome as a reference (GenBank accession number: NC034767.1). We performed de novo genome assemblies using Platanus v1.2.4 (Kajitani et al. 2014) with default parameters and *k*-mer lengths ranging from 45 to 99. A custom Perl script, plat.pl (Ohdera and Ryan 2018), was used to invoke the Platanus commands for assembly, scaffolding, and gap closing. We then used the suboptimal assemblies (*k*-mer=45, 64, 99) to construct artificial mate-pair libraries for five insert sizes (2,000, 3,000, 5,000, 7,000, 10,000) with MateMaker v1.0 (Ryan 2015b). SSPACE Standard v3.0 (Boetzer et al. 2011) was subsequently used to scaffold the optimal assembly (generated using *k*-mer=85) using the previously generated artificial mate-pair libraries. We removed contigs smaller than 200 bp for our RAD-seq analysis, however, the uploaded final assembly (https://doi.org/10.5061/dryad.c59zw3r40) still contains these reads. The commands and parameters used for the genome assembly are available in a github repository (Ketchum 2020). We checked completeness of the genome using CEGMA v2.5 (Parra et al. 2007) and BUSCO v2.01 (Simão et al. 2015) through the gVolante web server (Nishimura et al. 2017).

### Restriction Site-Associated DNA Sequencing and Data Processing

#### Sample Collection.

**Fig. 1.**
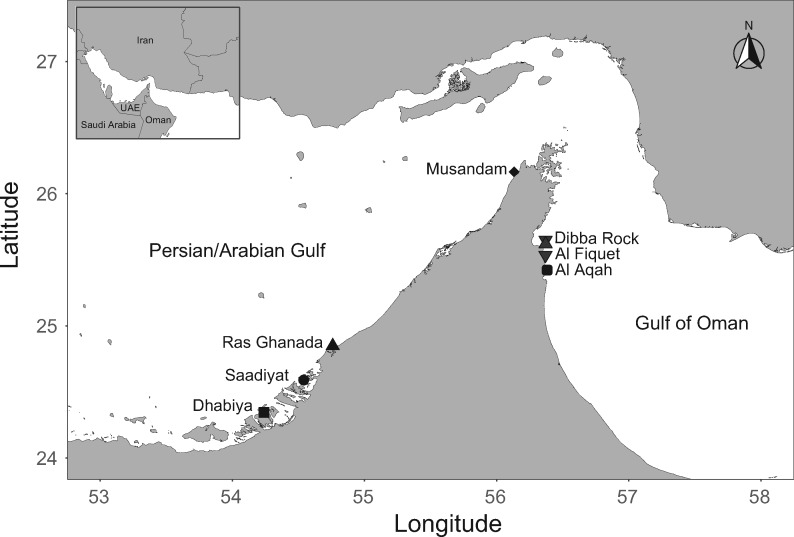
Map of the seven *E.* sp. *EZ* sampling locations.

Ten to fifteen *E.* sp. *EZ* individuals were collected between 2017 and 2018 from seven sites along the northeastern Arabian Peninsula, for a total of 94 samples (fig. 1 and supplementary table 1, Supplementary Material online). Four of the sites were in the environmentally extreme PAG and three of the sites were in the GO. Gonadal tissue samples were preserved in RNA*later*, and subsequently stored at −20 °C.

#### DNA Extraction and Sequencing.

DNA was extracted using the DNeasy Blood and Tissue Kit (Qiagen) following the manufacturer’s protocol for DNA extraction from tissues. DNA concentrations were normalized to 1 ng/μl for a total of 50 ng per reaction. Library preparation and sequencing of RAD markers were performed by Floragenex Inc. (Eugene, Oregon) using the restriction enzyme *sbfI* and Illumina 100-bp single-end sequencing.

#### Read Processing.

The dDocent pipeline (Puritz et al. 2014) was used for read mapping, SNP calling, and SNP filtering. First, raw reads were demultiplexed into separate files according to individual indices and quality filtered using “process_radtags” within *Stacks* v1.46 (flags: -e *sbfI* -c -q -r), which removes any reads with an uncalled base and discards reads with low-quality scores (Catchen et al. 2013). RADtags were aligned to the draft genome using BWA-mem v0.7.17 (Li and Durbin 2009; Li 2013) with default parameters. Samtools v1.7 (Li et al. 2009) was used to sort and filter out any alignments that had a mapping quality <30 and FreeBayes v1.1.0 (Garrison and Marth 2012) was used to call SNPs using default parameters.

#### SNP Filtering.

Preliminary filtering of variants was performed with VCFtools v0.1.14 (Danecek et al. 2011). We used the following parameters in the dDocent SNP filtering pipeline: 1) quality score ≥30; 2) minimum depth for a genotype call ≥3; 3) individuals with ≥50% missingness were removed; 4) a genotype call rate of 95% was applied across all individuals; 5) minimum mean depth ≥20; 6) population-specific call rate ≥90%; 7) minor allele frequency (MAF) ≥0.05; 8) removed loci with an allele balance ≤0.25 or ≥0.75; 9) removed loci above a mean depth of ≥475; and 10) kept SNPs that had frequencies that were not statistically different from Hardy–Weinberg equilibrium (cutoff=0.25, alpha=0.01, applied on a per population basis). After filtering, a total of 918 SNPs remained in 85 sea urchins (number of individuals per site: DH=11, SA=8, RG=11, MS=11, DB=15, AF=14, AA=15). This filtered VCF file was used for downstream analyses. The commands and parameters used in these analyses are available in a github repository (Ketchum 2020). 

#### Summary Statistics, Population Differentiation, and Structure.

Pairwise genetic differentiation (*F*_ST_) between populations and their significance was calculated in Arlequin v3.5.2.2 (Excoffier et al. 2005) with 10,000 permutations. Genetic diversity statistics within populations, including observed heterozygosity (*H*_O_), expected heterozygosity (*H*_E_), and the nucleotide diversity of variable sites (*P*i) were estimated using “populations” in *Stacks* v1.46.

We used STRUCTURE v2.3.4 (Pritchard et al. 2000), which implements a Bayesian clustering algorithm and ignores geographic proximity, to estimate the most likely number of genetic clusters. The number of clusters (*K*) was set from 1 to 10 with 20 independent runs for each fixed number of *K*. Each run included a burn-in period of 100,000 iterations, followed by 100,000 iterations of the Monte Carlo Markov Chain (MCMC) algorithm. The admixture model was run with correlated allele frequencies. To identify the most probable number of groups (*K*) that best fit the data, we used STRUCTURE HARVESTER (Earl and vonHoldt 2012), which implements the Evanno method to determine the optimal value of *K* depending on the Δ*K* value. The program CLUMPP v1.1.2 (Jakobsson and Rosenberg 2007) was used to align the 20 repetitions of the *K* value with the highest likelihood. These results were then visualized as bar plots using a custom R script.

To statistically test for population structure, we used principal component analysis (PCA) within the smartpca program in EIGENSOFT v6.0.1 (Patterson et al. 2006). We then used the twstats program within EIGENSOFT to perform a formal statistical test for population structure by calculating the significance of each eigenvector with a Tracey–Widom test.

#### Estimating Historical Relationships

Treemix v1.13 (Pickrell and Pritchard 2012) was used to understand historical patterns of gene flow between populations. Treemix leverages allele frequencies to generate a maximum likelihood tree for a set of populations and then connects branches in the tree with edges (or migration events) to explain excess covariance and improve model fit. The Al Aqah population was chosen as the outgroup because it is the most geographically distant from the PAG. The PAG is roughly 14,000 years old (Lambeck 1996) and we therefore hypothesized that the populations in the GO are ancestral to those in the PAG. Consideration should be taken when interpreting these results as it is possible that Al Aqah is not sufficiently genetically distinct from the other populations and accuracy has been shown to decrease when outgroups are not present in the data (Pickrell and Pritchard 2012). We ran Treemix for 0–10 migrations using the parameters -bootstrap -noss -k 500. Migration edges were plotted until 99.8% of the variance in ancestry between populations was explained by the model. The consistency of migration edges was visually evaluated by running Treemix with 30 total replicates for each added migration edge number. Further, each of the 30 replicates was run using a different, randomly generated seed. We present results from one seed that had the highest likelihood for each number of migration edges.

#### Detection of Loci under Putative Selection.

To identify outlier loci, we used a Mahalanobis distance-based approach in the R package *pcadapt* (Luu et al. 2017), which has been shown to be robust to a high degree of admixture and does not assume prior knowledge of population structure. Population structure was inferred using PCA, and putative outliers were detected with respect to how they relate to population structure. The number of principal components (*K*) was defined by running a PCA with *K *=* *1–20, and applying Cattell’s graphical rule (Cattell 1966) to the screeplot of eigenvalues to determine the optimal number of principal components, as recommended by Luu et al. 2017. Finally, we used the R package *qvalue* to generate a list of candidate outlier SNPs using the *q* value procedure at a false discovery rate (FDR) of α = 0.1 (meaning that 10% of the SNPs are expected to be false positives).

We performed a search using the Nucleotide Basic Local Alignment Search Tool (BlastN) on the genomic scaffolds which contained outlier loci against NCBI’s Nucleotide collection (nr/nt) database in order to functionally annotate the identified outliers. An *E* value cutoff of 10^−8^ was used and only outlier SNPs which were within 1 kb of the BLAST hit were retained.

## Results

### Draft Genome

The optimal assembly (*k*-mer=85) resulted in a genome assembly with 4,487,317 scaffolds, measuring a total of 1.59 Gb. The assembly had an N50 of 1,006 bp and a mean coverage of ∼27×. We recovered 60% (16% complete and 44% partial) of the core eukaryotic genes and 75% (37% complete and 38% partial) of the core metazoan genes with CEGMA and BUSCO, respectively. The low recovery rates for conserved genes in the *E.* sp. *EZ* genome are due to the fragmented nature of the genome, which is likely a direct consequence of high heterozygosity and repeat content (25% of the genome comprised repeat regions). The estimated genome size was 609 Mb, the heterozygosity rate was 4.54%, and the duplication levels were 0.6%.

### RAD Sequencing

RAD sequencing of 94 *E.* sp. *EZ* individuals resulted in 347,439,950 total sequences, of which 287,973,771 (82.9%) were retained after initial quality filtering steps. Of the 59,446,179 discarded reads, 0.05%, 1.6%, and 15.4%, were discarded due to low-quality, ambiguous RAD-tags, and ambiguous barcodes, respectively. After mapping RADtags to the draft genome assembly and filtering for mapping quality, 378,775 SNPs were called. After stringent SNP filtering, 918 SNPs from 85 individuals across seven populations were kept. These 918 SNPs were used in downstream analyses unless a program did not allow triallelic SNPs, in which case we used a reduced VCF file containing 901 SNPs. This low SNP retention rate was likely due to extremely high heterozygosity and an abundance of repeat regions in the *E*. sp. *EZ* genome (Gautier et al. 2013).

#### Population Genetic Diversity and Structure.

Estimates of *H*_O_ and *H*_E_ across 901 SNPs were consistent across the seven sampling sites (*H*_O_=0.2076–0.2343, *H*_E_=0.2376–0.2572, table 1). Nucleotide diversity ranged from 0.2491 to 0.2670 and was similar to *H*_E._

**Table 1:** Summary of Genetic Diversity Statistics for Seven Populations of *E.* sp. *EZ*

Pop ID	Location	Variant Sites	% Polymorphic Loci	Num Indv	*H*_O_	*H*_E_	*P*i
Dhabiya	S-PAG	900	89.5556	10.8278	0.2266	0.2472	0.2592
Saadiyat	S-PAG	901	89.1232	10.8169	0.2076	0.2376	0.2491
Ras Ghanada	S-PAG	901	94.1176	14.7714	0.2151	0.2471	0.2558
Musandam	N-PAG	901	94.4506	13.8224	0.2343	0.2572	0.2670
Dibba Rock	GO	900	91.5556	10.7744	0.2163	0.2499	0.2621
Al Fiquet	GO	900	84.2222	7.9744	0.2172	0.2391	0.2552
Al Aqah	GO	901	95.3385	14.8058	0.2323	0.2554	0.2649

Note.—S-PAG, Southern PAG; N-PAG, Northern PAG; GO, Gulf of Oman; Variant sites, number of total SNPs; % Polymorphic loci, proportion of SNPs in this population; Num Indv, mean number of individuals per locus in this population; *H*_O_, mean observed heterozygosity per population; *H*_E_, mean expected heterozygosity per population; *P*i, mean nucleotide diversity.

The only significant pairwise *F*_ST_ values were found when comparing populations from inside the PAG to those in the GO (table 2). The highest significant *F*_ST_ values (0.02514, *P* value = 0.00000 and 0.02189, *P* value = 0.00000) were found when comparing Al Fiquet (GO) to Saadiyat (S-PAG) and Dhabiya (S-PAG), respectively; these PAG sites are the most geographically distant from the GO. When all samples in each respective Gulf were pooled, the *F*_ST_ between the PAG and the GO was 0.0057.

**Table 2:** *F*_ST_ Values from 918 SNPs across Seven Populations of *E.* sp. *EZ*

	Dhabiya	Saadiyat	Ras Ghanada	Musandam	Dibba Rock	Al Fiquet	Al Aqah
Dhabiya	—	—	—	—	—	—	—
Saadiyat	0.01241	—	—	—	—	—	—
Ras Ghanada	0.01208	0.01114	—	—	—	—	—
Musandam	0.01168	0.00665	0.00658	—	—	—	—
Dibba Rock	**0.01479**	**0.01931**	0.0062	0.00906	—	—	—
Al Fiquet	**0.02189**	**0.02514**	**0.01529**	**0.01496**	0.00625	—	—
Al Aqah	**0.01578**	**0.01996**	**0.01244**	**0.01349**	0.00275	0.00251	—

Note.—Values in bold were significant (*P* < 0.05).

**Fig. 2.**
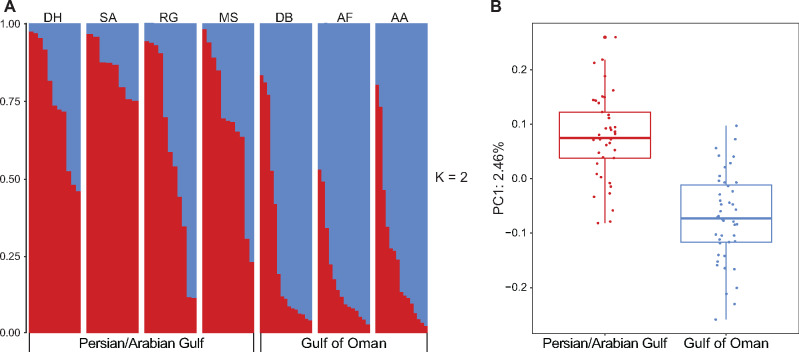
(*A*) Plot of the individual ancestry inference for *K *=* *2 based on 918 loci. The population abbreviations are as follows: DH, Dhabiya; SA, Saadiyat; RG, Ras Ghanada; MS, Musandam; DB, Dibba Rock; AF, Al Fiquet; AA, Al Aqah. (*B*) Box plot of eigenvalues for 85 individuals explained by principal component one, generated in the smartpca package.

To further characterize population structure, we used STRUCTURE, smartpca, and a Tracey–Widom test. The Evanno method, which evaluates the second-order rate of change of the likelihood function with respect to Δ*K*, identified *K *=* *2 (with Δ*K*=5.2236, see supplementary fig. 1, Supplementary Material online) as the optimum number of populations from the STRUCTURE output (fig. 2; *K *=* *3 and *K *=* *4 are available in supplementary fig. 2, Supplementary Material online). The STRUCTURE plots showed population structure between the PAG and the GO, although a high degree of admixture resulted in all individuals with identities corresponding to both seas. We used smartpca to generate a principal component analysis (PCA) plot and we applied Cattell’s graphical rule (Cattell 1966) to the associated scree plots, which indicated that the optimal number of principal components was one (fig. 2). In other words, the majority of the variation in the data was explained by the first principal component and all subsequent axes only served to explain random variation. As the Evanno method cannot formally test for *K *=* *1, we used the Tracey–Widom test to calculate the significance of eigenvectors (generated in smartpca) and subsequently, the number of populations within the data set. We found that only the first eigenvector was significant (*P* value=3.37e-06) and explained 2.46% of the total genetic variation. All population structure analyses showed a slight degree of population differentiation.

#### Historical Relationships.

**Fig. 3.**
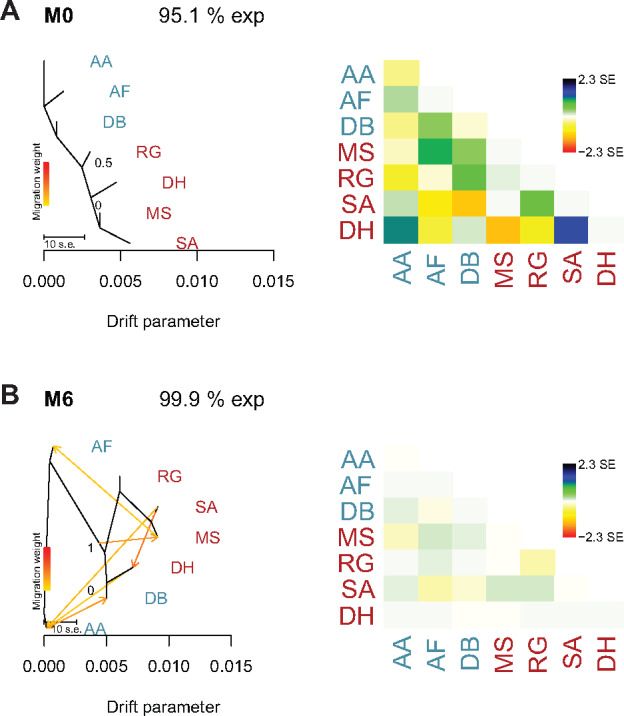
Phylogenetic network of the inferred relationships between seven populations of *E*. sp. *EZ*. The population abbreviations are: DH, Dhabiya; SA, Saadiyat; RG, Ras Ghanada; MS, Musandam; DB, Dibba Rock; AF, Al Fiquet; AA, Al Aqah. Population abbreviations were colored based on their Gulf of origin (PAG, red; GO, blue). Migration edges are colored according to percent ancestry received from the donor population and SE represents the standard error of migration rates. (*A*) M0 represents a phylogram with no migration edges. (*B*) M6 contains six migration edges. Next to each phylogenetic network are the corresponding residual plots.

We ran TreeMix with 85 sea urchin samples from seven populations to identify patterns of divergence and add migration edges to the phylogenetic model. The proportion of variance began to asymptote at 0.999 when six migration edges were fit (supplementary fig. 3, Supplementary Material online). The consistency of these runs were evaluated using 30 independent runs of TreeMix with all ten migration edges. Across all iterations, 95.1% of the total variance was explained by the graph model without any migration edges. The phylogenetic tree shows separation between the two seas, recapitulating the results found through population structure analyses (fig. 3). The first migration edge showed a migration event from Saadiyat (SA) to Dhabiya (DH), which are both located in the southern PAG (fig. 3). This result was consistent across all 30 replicates. Residual plots showed that as more migration edges were added, the proportion of variance in relatedness between populations explained by the models continued to increase. At six migration edges, there were more vectors moving from the PAG into the GO than from the GO into the PAG (the phylogenetic network at six migration edges was consistent across all 30 replicates). However, there were migration edges moving both in and out of the PAG, consistent with previous results that reveal a high degree of admixture between these seas (all migration events shown in supplementary fig. 4, Supplementary Material online).

#### Candidate Loci under Selection.

Outlier detection in *pcadapt* was performed by retaining loci correlated with the first principal component axis after a 10% FDR correction. Of the 918 SNPs generated across 85 individuals, we identified nine candidate outliers. Of these nine outliers, we eliminated the loci that did not have a clear BLAST match (*E* values cut off of 10^−8^), and those whose associated genomic scaffold had a query length <5,000 bp. This resulted in one outlier on scaffold 0012050 (length of query: 7,820 bp) that was located in an *E.* sp. *EZ COLP5α* (5α collagen-like chain) (Exposito et al. 1995) gene, which has a clear ortholog in *Strongylocentrotus purpuratus* (the purple sea urchin; Accession number: AC165428.1). In adult sea urchins, this gene is expressed in mineralized regions and in the adult mutable collagenous tissues (Cluzel et al. 2001). However, the exact function of this gene has not been well characterized (Exposito et al. 1995).

## Discussion

We sequenced and assembled a draft genome for the sea urchin *E.* sp. *EZ* and generated a population genomic data set consisting of seven populations from two environmentally distinct seas. With these genomic tools, we analyzed population dynamics of individuals living across dramatically divergent thermal and salinity environments. This study contributes to the growing body of literature characterizing population dynamics in environmentally extreme marine systems, and represents the first genome-wide investigation of a sea urchin from northeastern Arabia. Our findings are relevant to predicting how species will respond to future and ongoing climate change.

*Echinometra* is a pantropical genus with geographical distributions across the Indo-Pacific, Caribbean, and Atlantic. *Echinometra* is widely studied and recognized for their distinct patterns of population structure and speciation dynamics (McCartney et al. 2000; Landry et al. 2003; Lessios 2006; Bronstein and Loya 2013). The availability of the genomic-level sequence data will be a useful tool to explore the unique ecological and reproductive dynamics of this genus as well as a tool for comparative genomics with other sea urchins, as there are only a few sea urchin genomes available (Cameron et al. 2015). Until a recently published mitochondrial genome became available (Ketchum et al. 2018), *E*. sp. *EZ* was misidentified as *E. mathaei*, highlighting the importance of genomic resources for taxonomic identification and associated studies.

A notable characteristic of the *E*. sp. *EZ* genome was the high frequency of polymorphisms (the estimated heterozygosity was ∼4.5%). This is likely a result of either large population size or an elevated mutation rate. In the sea squirt, *Ciona savignyi*, high heterozygosity (4.49%) was shown to be driven by a large effective population size, not elevated mutation rates (Small et al. 2007). This level of heterozygosity is also comparable with the *Strongylocentrotus purpuratus* genome, which required the sequencing of large BAC clones to parse haplotypes (Sodergren et al. 2006). The high genomic variation results in a challenging genome assembly as it is difficult to distinguish between reads that are from duplicated but diverged sections of the genome or highly heterozygous homologs (Sodergren et al. 2006). This problem is further aggravated by repeat sequences and short-read sequencing data. These variables resulted in a genome assembly that was too highly fragmented to perform gene annotations. However, our assembly was a valuable resource for our RAD-seq analyses.

Oceanographic circulation patterns, selective pressures exerted by temperature and salinity extremes, effective population sizes, and dispersal capabilities are all factors that may govern population structure and gene flow. It is often assumed that the life history traits of many marine invertebrates (i.e., long pelagic larval durations and large effective population sizes) should result in a lack of genetic structuring between geographically distant populations (Waples 1998; Casteleyn et al. 2009). Modern coastlines in the PAG were formed only ∼6,000 years ago following the Holocene transgression (Lambeck 1996) and our two most distant sites are ∼500 km apart. Therefore, it would be reasonable to assume that marine organisms inhabiting the PAG and GO may represent one panmictic population. However, our data suggest weak but significant population structuring between the two seas.

The *F*_ST_ results shown in table 2 indicate population differentiation between the PAG and the GO sites. Indeed, the only significant *F*_ST_ values were found when comparing sites within the PAG to sites within the GO. The only *F*_ST_ values that were not significant between the two seas were found when comparing Dibba Rock to Musandam and to Ras Ghanada (the two PAG sites closest to GO). No significant *F*_ST_ values were found when comparing between sites within the same sea. These findings are congruent with studies on other marine organisms in the region, which also describe significant population differentiation between the two seas. One study on the sea urchin *Diadema setosum* used mitochondrial DNA to investigate population structure around the Arabian Peninsula and found that *F*_ST_=0.05 (Lessios et al. 2001). A study on the coral *Platygyra daedalea* analyzed the ITS region and found *F*_ST_ values ranging from 0.051 to 0.29 (Smith et al. 2017a). Finally, a study on the yellowbar angelfish, *Pomacanthus maculosus*, generated a SNP data set with 10,225 SNPs and found that the *F*_ST_ between the two seas was 0.015 (Torquato et al. 2019). The *F*_ST_ values calculated in these studies all indicate more population structure than what was found in *E.* sp. *EZ* with the exception of some pairwise comparisons between specific sampling locations. The differences in the magnitude of values may be due to sequencing different gene regions and using different sequencing approaches. Although *F*_ST_ calculations were comparatively lower in *E*. sp. *EZ*, despite the fact that urchins are expected to be a high gene flow species (e.g., large population size, high larval dispersal capabilities, and reproductive output), we were still able to detect weak but significant population differentiation. Together, these studies support a hypothesis of a consistent genetic break for many species between the PAG and GO.

Through multiple analyses, we revealed the presence of two populations of *E.* sp. *EZ*, each corresponding to their respective Gulf. Interestingly, the STRUCTURE analysis revealed that assignment probabilities of individuals varied greatly within and between populations. For example, in every collection site in the PAG except Saadiyat, there are some individuals whose assignment probabilities more closely associate with the GO than the PAG. This same subtle pattern can also be seen for individuals from the GO whose assignment probabilities more closely resemble individuals from the PAG. These results are in contrast to a similar study on the coral *P. daedalea* by Howells et al. 2016 where one site within the PAG and one site within the GO were sampled. They also found that the most likely number of populations was two. However, the assignment probabilities of individuals were more clearly differentiated and there was little evidence of admixture. This could be a result of different larval characteristics (e.g., lecithotrophic vs. planktotrophic larvae and associated differences in PLD) as the degree of genetic exchange and subsequent population structure in these two species relies on larval migration.

The patterns of admixture in *E*. sp. *EZ* between the two seas could be a result of a high degree of unidirectional or biased migration events. Oceanographic models have shown reduced mixing between the seas through subsurface outflow that prevents the transport of buoyant larvae, as well as long residence times of ∼1–3 years for seawater in the PAG (Alosairi et al. 2011). However, our preliminary findings suggest that migration occurs bidirectionally between seas, although there were more migration vectors moving from the PAG to the GO. Alternatively, this population structure could be a result of genetic drift or the extreme environmental conditions of the PAG, which may act as a selective pressure on urchins and other species. Future research should employ demographic models to explore these hypotheses as well as explore other possible demographic events (e.g., range expansions, founder events, and population bottlenecks) which may have or may continue to shape allele frequency patterns between populations.

Our outlier analysis was preliminary and only resulted in nine significant outliers under putative selection. We could only functionally annotate one of these outliers; *COLP5α.* It is currently unclear what the exact role of this gene is in sea urchins but it has been implicated in collagen formation. Collagen genes have been shown to respond transcriptionally to thermal stress in other marine invertebrates (DeSalvo et al. 2010; Kenkel et al. 2013). Further studies are warranted to generate a more suitable data set for investigating genes under natural selection and to better understand the main drivers of this population differentiation.

Our study contributes to recent efforts to characterize the population dynamics of organisms across extreme environmental gradients. The most striking result from this analysis was the presence of population structure given that the young age of the PAG, the dispersal capability of *E*. sp. *EZ*, and the large effective population sizes, should all act to homogenize population differentiation. The RAD-seq dataset and *E.* sp. *EZ* draft genome assembly presented here will provide a platform for future studies on this ecologically important and understudied species.

## Supplementary Material

evaa150_Supplementary_Data

## Notes

**Data deposition: This project has been deposited at NCBI Sequence Read Archive Database under BioProject ID PRJNA613076 and Dryad at https://doi.org/10.5061/dryad.c59zw3r40.**
