# Supplementary material for: Population Genomic Analyses of the Sea Urchin Echinometra sp. EZ across an Extreme Environmental Gradient
Source: Genome Biol Evol. 2020 Jul 22;12(10):1819–29. doi: 10.1093/gbe/evaa150 (PMC7594579; doi:10.1093/gbe/evaa150)
Supplement: evaa150_Supplementary_Data [file evaa150_supplementary_data.docx]

**Supplemental Data:**

**Supplemental Table 1**. Sampling site names and ID, as well as the coordinates of sampling sites.

AA

BA

**Supplemental Figure 1.** STRUCTURE HARVESTER output from STRUCTURE analysis which reveals the most probable number of populations. **A.** Delta *K* is related to the second order rate of change of the log probability of data with respect to the number of clusters and is a good predictor of the number of populations (*K* = 2). **B.** Log probability of data L(*K*) as a function of K.

**Supplemental Figure 2:** STRUCTURE plots of the individual ancestry inference for K = 3 and K = 4 based on all 918 loci. The population abbreviations are as follows: DH = Dhabiya, SA = Saadiyat, RG = Ras Ghanada, MS = Musandam, DB = Dibba Rock, AF = Al Fiquet, and AA = Al Aqah.

**Supplemental Figure 3:** Box plot showing the fraction of variance in relatedness between populations accounted for by phylogenetic models with 0 to 10 migration edges. The fraction begins to asymptote near 0.998 at six migration edges.

**Supplemental Figure 4:** Phylogenetic network of the inferred relationships between seven populations of *E*. sp. *EZ*. The population abbreviations are: DH = Dhabiya, SA = Saadiyat, RG = Ras Ghanada, MS = Musandam, DB = Dibba Rock, AF = Al Fiquet, and AA = Al Aqah. Population abbreviations were colored based on their Gulf of origin (PAG = red, GO = blue) and s.e. represents the standard error of migration rates. Migration edges are colored according to percent ancestry received from the donor population. M0 represents a phylogram with no migration edges and each increasing number (e.g., M1-6) represents an additional migration edge. Next to each phylogenetic network are the corresponding residual plots.

**Supplemental Figure 5.** As our analyses does not account for linkage disequilibrium (LD), we tested the impact of LD on our dataset by using the *–-indep-pairwise* command in Plink (Purcell et al. 2007) with a 10kb window, a step size of 1, and a pairwise r^2^ threshold of 0.2. The LD-pruned SNP dataset containing 691 SNPs in linkage equilibrium were analyzed in smartpca to verify that population structure was not a result of LD. We used a Tracey-Widom test to calculate the significance of each eigenvector and found that PC1 was significant with a *P*-value = 0.000727, indicating that there are two populations in the dataset.
